# On the Limits of the Data Economy: The Case of Autonomous Vehicles

**DOI:** 10.1007/s11948-025-00540-5

**Published:** 2025-07-01

**Authors:** Björn Lundgren

**Affiliations:** 1 Centre for Philosophy and AI Research, Friedrich-Alexander-Universität Erlangen-Nürnberg, Erlangen, Germany; 2 Institute for Futures Studies, Stockholm, Sweden

**Keywords:** Data economy, Surveillance capitalism, Privacy, Anonymity, Ability to be anonymous, Risk, Informational risks, Autonomous vehicles

## Introduction

Autonomous, automated, or self-driving vehicles (AVs) are already on our streets.^1^ For policy purposes we should prepare for their broad arrival within the reasonably near future.^2^ Despite this, many of the fundamental ethical questions, which require a response to develop ethically appropriate policies for AVs, remain unanswered.^3^ While large parts of the literature have focused on the ethics of unavoidable crashes and issues relating to responsibility,^4^ many have also recognized that AVs raise substantial questions relating to privacy and information ethics.^5^ Nevertheless, there are relatively few substantial treatments on the topics of privacy and information ethics as far as AVs are concerned.^6^

Inevitably, AVs need to have access to information about their surroundings, travel paths, location, destination, and time, which raises concerns regarding privacy, surveillance, anonymity, and data protection. While some have focused on basic requirements for information protection (encryption, deletion, and so forth),^7^ this article aims to go beyond basic requirements or the trade-off between safety and data protection. Specifically, the article will address the question of whether it is permissible to collect information for non-safety purposes or to re-use information collected for the purpose of safe transportation, for non-safety-related purposes (e.g., for purely commercial purposes or state surveillance).^8^ That is, the article contributes to the overarching question of whether it would be permissible to expand the data economy into the transport sector (i.e., further than what has already happened through services such as Google Maps).^9^

In this paper, I am setting aside the question of whether some information needs to be retained because of legal auditing or other ethically motivated requirements. What I am interested in is two questions. First, granted that some data *must* be collected, created, processed, and used for safety purposes (or due to other ethical requirements), is it permissible to further use or distribute that data for other—purely commercial or state surveillance—purposes? This question is simply about double-dipping of data. Second, AVs make it technically possible to collect, create, process, and use large sets of data beyond that data that would be required to fulfil the safety or other moral demands for which it was allegedly gleaned. Is it permissible to collect, create, process, and use such data? I argue that in both of these cases, it is not. (*N.B*., “data” and “information” will henceforth be used interchangeably, even if there is a meaningful technical difference between these terms; moreover, to simplify I often use formulations such as *using data* or *collecting data* to indicate *collecting*,* creating*,* processing*,* using*,* and distributing data*).

I will not attempt to define the precise limits of safety purposes; I believe it will be sufficiently clear what I mean for my arguments to be meaningful. Setting the precise limit of what is needed for safety purposes or what information that is otherwise ethically permissible to use would be important if I had intended, which I have not, to address the question of how we should solve the trade-off between the information needed for safety (and other) purposes and the potentially detrimental effects of using that information. Instead, I remain agnostic as to the fundamental question of whether AVs are ethically permissible to use. That is, what I address is simply the question of what use of information should be permitted granted that AVs are permissible (and hence must collect some data for safety purposes). Put otherwise, what I am asking is basically whether the rules (or the partial absence thereof) for the Internet-based economy should apply to transportation as well.

The remainder of the paper is structured as follows. Next, in the second section, I briefly discuss why the use or collection of data matters. This lays a foundation for the upcoming analysis since the ethical restrictions on the use or collection of data depend on the risks involved. In the third section, I turn to address the question of whether it is permissible to collect and use data from non-users (of AVs) for non-safety purposes. In the fourth section, I turn to the users of AVs to address the same question. In both sections, my arguments rely on a discussion of informed consent and ideas from the ethics of risk. In the last section, I summarize my arguments and comment on the broader consequences of the arguments.

Finally, I use a novel method to argue for my conclusion in this paper. While the concerns that I address could be approached in different ways, my main arguments rely on analyzing these problems in terms of balancing interests (*mutatis mutandis* for other normative theories). In resolving these balancing acts, I will primarily treat these as questions of *risk imposition*, relying on the work by Sven Ove Hansson (according to which we have an overridable right against risks). Although this is a novel approach in the given context, it should be noted that privacy scholars—some of whom also rely on the work of Hansson—have recently linked the philosophy of risk to the debate on the right to privacy.^10^ Moreover, to complement this normative perspective I will also briefly sketch how purely consequentialist theories may be applied to reach similar conclusions.

## Why Should we Worry About Data Protection and What Does it Have to do with AVs?

Surveillance capitalism is already here, and many think it is bad news. The philosophical literature includes a broad set of concerns: that others can track us through varying contexts;^11^ that others can reduce *our ability to be anonymous* (in some given context),^12^ which also implies an increased ability for others to infer information based on our communications or actions;^13^ that collected or inferred information can be used to manipulate or harm us;^14^ that our individuality becomes reduced to data points;^15^ that collected or inferred information is unequally distributed so that only a few limited agents gain the informational benefits, which also results in a skewed power balance and is detrimental to our ability to act as free agents on the marketplace;^16^ and that the risk of informational power abuses through surveillance ultimately threatens individual liberties as well as our democratic system more broadly.^17^

One may ask what role AVs have in such dystopian—yet realistic—worries; the answer is simple: AVs have the potential to contribute to mass surveillance by transforming the transportation economy into a surveillance system with each vehicle equipped with cameras or other visualization systems.^18^ If individual AV systems are jointly combined—which may be motivated purely on safety grounds—then we will have a moving surveillance system on our streets; a substantial change from the current “possib[ility] to travel anonymously.”^19^ The jointly collected data would allow the tracking of both users and non-users (e.g., pedestrians) in any sufficiently traffic-dense area. Such tracking would, in turn, reveal patterns, which could be used to predict further sensitive information.^20^ Just as a smartphone quickly learns where you work and where you live, because of time-stamped GPS locations (or Wi-Fi-access and so forth), this information could be used to map all your travel-related patterns. Combining these data, one could see who your friends are; if you have a secret lover; which religious or political events you frequent; where you shop, work out, or have your coffee; and how often you visit bars, the doctor, or other establishments that may be privacy-sensitive. Again, such information could, in turn, be aggregated to predict or infer further information.^21^ The basic problem is that the distinction between sensitive and insensitive information would practically collapse, such that highly sensitive information could be predicted based on seemingly limited datasets.^22^ This should is worrisome, because if more information is collected, then more powerful prediction models can be constructed. Simply put, the more you know about an individual, the easier it is to adapt information manipulation to a singular individual. Hence, if we are worried about the influence of misinformation and the manipulation of individuals or collectives, then we should be worried about increased surveillance.^23^

The risks discussed above are not merely the result of the AV’s visualization system, information could also be collected from GPS data, timestamps, travel data, passenger information, applications that include surveillance of in-vehicle behavior, smartphones linked to the AV (inside or in the vicinity of the AV), and other potential data-sharing or data-harvesting functions. Moreover, there are various examples in the literature on how, for example, geo-positional data can be used for commercial purposes.^24^ There are also reports of abuse of collected data, such as Tesla users’ video recordings being saved by Tesla employees.^25^

From this brief overview, it should hopefully be clear that what is at stake is quite substantial and important: what is at stake is not only our individual informational privacy but our way of life and the function of liberal democracy. Since more information and data usage implies a greater risk towards these fundamental human values and a successful democratic system, we need to take seriously the substantial added risks that AVs create through expanding surveillance capitalism.

## Requiring Maximal Data Protection for Non-Users

In this section, I will discuss whether non-users’ data and information can be used or collected for other purposes. To simplify, I will introduce some terminology. I will use the term *maximal data protection policy* (‘MDPP’) to refer to whatever data policy satisfies the demands on limiting data usage to safety purposes or other permissible purposes of data collection (such a policy may include requirements such as encryption, deletion, and minimization of data collection).^26^ Moreover, I will talk of *AV service providers* in a broad sense as the group of agents (including institutional agents) that are in control of AVs’ functionality (which may include manufacturers, AV taxi providers, regulators, and so forth). That is, I will set aside the question of how responsibility is distributed between different parties so that I can focus on the question of permissible data usage.

In light of the worries about surveillance and data collection, the easiest question to address is what the ethical limitations are for AV services to collect and use data about non-users (i.e., individuals who are not currently using the AV services). *Prima facie* the response seems simple: AV services have no right to use or collect data from non-users for non-safety purposes because they have no consent agreement from them and no such consent agreement can be established.

However, the above argument could potentially make any ordinary form of transportation practically impossible since all transportation requires visual access to the nearby environment. Yet, the latter claim seems ridiculous because it seems *prima facie* evident that we have a *pro tanto* right to transport ourselves using vehicles. That is, if there is any absolute restriction on such usage it is likely grounded on arguments related to the climate, environment, or health; setting all such other considerations aside, it should be clear that the fact that a driver sees individuals in their surroundings is not a sufficient reason to forbid such forms of transportation—on the contrary, they have a responsibility to look out for them. If such data is collected, protected, and deleted when it no longer needs to be stored, it seems to matter little if a human or a machine performs the data collection. In fact, setting aside the security risks, it seems plausible to think that if sufficient data protections are in place (encryptions, deletion, and so forth), then privacy and other individual considerations may be better preserved if the data collection is done by a machine rather than a human.

While the right to consent is standard in information transfers, the rights involved are sometimes overridden (e.g., in some medical research). To understand when it is permissible to use information—without explicit consent—and when it is impermissible, I will reformulate the problem of consent to a problem of informational risk-taking. That is, using principles from the ethics of risk I will explain the difference between the *prima facie* permissibility to collect data for safety purposes and the impermissibility of further using that information for other purposes (i.e., commercial benefits or mass surveillance). According to Sven Ove Hansson, we have *an overridable right against risk exposure*. The conditions for overriding the right are as follows:

Exposure of a person to a risk is acceptable if and only if this exposure is part of an equitable social system of risk-taking that works to her advantage.^27^

Although these conditions—as Hansson recognizes—warrant *further* explication, they will suffice for this article. Indeed, although determining the precise distinction between what is and what is not equitable would be essential for *some* analyses, such precision will not be needed for the purpose of my argument as the normative balancing act is sufficiently clear to be fully compatible with vague or otherwise imprecise conceptions of the underlying normative concepts.^28^ To simplify, let me begin by noting that it should be clear that while a system of transportation can work to everyone’s advantage (as Hansson exemplifies), a system of mass-transport-surveillance would not. Of course, the question for non-users is not whether they benefit from what they are not using, but if that risk can be traded against another risk in a system that is equitable. Before turning to the main issue, let us first address the simplest case: A non-user of AV services can benefit from their existence, most obviously, because for most people the property of being a non-user is a temporary contingency; in most cases, whoever is a non-user of the system, at a given time, will be a user of the system at some other time. These non-users will benefit directly through using the same benefits provided to the users, but at another moment in time. Nevertheless, there are arguably some non-users that will never themselves make use of an AV, just as there are people who do not use certain types of vehicles today. This may hold even in the future when such services are the major means of transportation (having replaced cars and commercial road vehicles, trains, and perhaps even airplanes). However, most of these non-users will benefit from the transportation system to get access to goods or other societal services. We can set aside these contingent non-users, as I will address the question of users in the next section. However, for the individuals that remain fully outside that system, we have to analyze whether the system of risk-trading is equitable. For example, my right to use an AV (with the risk of harming a non-user) can be motivated because non-users can perform other risk-taking acts, as long as such risk-taking is part of an equitable social system of risk-taking that works to everyone’s advantage.

While the standard use of AVs should be permissible based on the above type of reasoning, the same kind of reasoning would arguably forbid the double-dipping of data usage (i.e., using data for non-safety purposes). Here it is illustrative to consider a standard problem in the ethics of risk: the asymmetrical distribution of risks and benefits. That is, there is often a non-equitable distribution of risks and benefits such that some people are exposed to the risks caused by a certain action and another group of people reaps the benefits of that action. What I just have said may seem to turn on the question of what precisely we take to be non-equitable. However, my argument will henceforth rely on cases that should be considered clear-cut cases that do not require an extensive theoretical analysis of borderline cases and so forth. Indeed, it should be clear that the type of data and data usage that I am concerned with here, which I discussed in the previous section, are subject to this asymmetry problem. That is, data usage by commercial parties (or by some other institutional agents) creates benefits for them, while potentially being detrimental to non-users. Because of this basic asymmetry, we can *prima facie* conclude that the system, in isolation, is non-equitable.

However, for the *prima facie* conclusion to be—all-things-considered—applicable we must broaden the analysis. Arguably, this hangs on two questions. First, does the risk-taking itself generate sufficient benefits to motivate it? Second, if not, then can the risk-taking be part of an equitable system of risk-taking that works to everyone’s advantage? In response to the first question, one may note that there is a potential trickle-down benefit created by the benefits garnered by commercial parties. Nevertheless, even if there are trickle-down benefits, they would likely benefit non-users to a lesser degree. For example, we can imagine that AV services would offer discounts if users were to give up some control of their information.^29^ While such systems might generate benefits for the economy overall, and hence also benefit non-users, the risks of information collection established in the previous section would obviously be much more substantial. This would put individuals’ liberty and privacy, democratic norms, and the democratic system at risk. We can see that this type of reasoning can also be motivated without concern for the non-users’ rights (e.g., based on democratic risks).

However, one may attempt to resist the above argument by arguing that non-users could gain benefits through surveillance. For example, AV services have the potential to make a neighborhood safer through surveillance. But that is precisely the type of consideration that can be satisfied without mass surveillance and commercial double-dipping of data. Such concerns are potential safety concerns (if we understand safety in the broader sense that I mentioned in the Introduction). For example, if a crime has been committed, one should—as would be standard for other available surveillance systems—allow for limited usage of such information for criminal investigations and legal proceedings.

Another alternative is to consider the idea that we should allow everyone to use the collected data. However, even if all such data were to be openly accessible and even if we were to set aside all of the problems of such extreme data transparency,^30^ the risks and benefits would still be asymmetrical because individuals with more resources (i.e., knowledge of the system, economic resources, and so forth) would possess a greater ability to both avoid the implicit mass surveillance of the system and benefit from the available data (i.e., it would require technical savvy and resources, which are possessed by only a few). Hence, the benefits and risks would still be asymmetrical. Therefore, it is difficult to see what kind of benefits (beyond safety) an AV service could provide for non-users that would motivate going beyond an MDPP.

Turning to the question of the potential for equitable risk-trading, it seems difficult to see what kind of risk-trading system would be beneficial if increased surveillance were to be part of that system. Indeed, if I am correct about the value-weights in the discussion under consideration, then a risk-trading system that allows these risks would be a system that would increase the societal risks in general. Given the meager benefits that the system potentially could provide, it is difficult to see how that could be to the benefit of all. Keep in mind that achieving equity will be difficult given the asymmetrical risk distributions.

The argument just presented seemingly relies on principles that potentially conflict with various common consequentialist theories, such as classical utilitarianism (because they are concerned with the distribution of benefits, not maximization), but we can see that the argument holds from a classical utilitarian perspective as well, because the risks of implementing a new form of surveillance system outweigh its potential benefits. The risks of abuse are not insubstantial and the results would be so severe that we ought to impose restrictions on purely utilitarian grounds. Consider the fact that AI technology is already being used in non-democratic nations for the surveillance of the population.^31^ That is, we ought to, as a matter of increasing the chances of achieving the most utility or the best outcomes, avoid mass surveillance systems, because of the extreme risks of abuse.^32^ Some might object to this argument and suggest that by sharing a limited set of data, we need not worry. However, as I noted in the previous section, the prowess of information aggregation technologies does not allow us to maintain the distinction between sensitive and non-sensitive information because of the ability to infer sensitive information based on non-sensitive information. Hence, we need to impose an MDPP to protect against all such options.

Based on what has already been said, we can conclude that AV service providers must impose an MDPP for non-users. In summation, since non-users cannot offer consent, I will reformulate the problem in terms of an overridable right—on the part of the non-users—against informational risk exposure. Since the conditions under which their data is used are *not* part of a sufficiently equitable social system for trading risks, the right cannot be overridden. Lastly, because the imbalance is, arguably, so extreme, these arguments also hold on purely consequentialist grounds.

## Requiring Maximal Data Protection for Users

In the previous sections, I argued that AV service providers must enact an MDPP for non-users. In this section, I will argue that the AV service providers must also enact an MDPP for users. Part of the argument I presented in the previous section establishes that we have consequence-based arguments for preferring an MDPP. The commercial benefits of using data freely do not seem to outweigh the risks previously discussed in the second section. That seems to hold irrespective of whether we consider users or non-users. However, many ethicists would think that we have a right to go into individual agreements, despite its potential costs. That is, *prime facie*, users should have a *pro tanto* right to give consent to a more minimalistic data protection policy, for *their data*. I now turn to disqualifying this right.

To start with, the *prima facie* presumption mentioned above is based on a false dichotomy: the idea that information about oneself can be fully separated from information about others. To see why this is misleading, consider, for example, a model by Michal Kosinski et al., which aims to predict a variety of potentially sensitive information (such as political or religious leanings, race, religion, sexuality, and so forth), using only what people have liked on Facebook as a model input (at the time the model was made, *Facebook likes* consisted merely of a thumbs-up on a given social media post). It is only possible to construct that model by using available data, but the available data of *some* users then makes it possible to infer new information about *other* users. That is, by sharing information about *ourselves* we indirectly share statistical information about others as well. If we cannot consent to share information about others, there seems that there must be some restrictions on sharing information about ourselves as well.

An illustrative example of the above problem is the case when a person, through a consent, share’s their DNA with a commercial interest, giving them various broad usage rights. People are currently entering such agreements to get analyses of genetic risks or to investigate their ancestry. Although an individual’s DNA is *individual* and hence not identical to anyone else’s DNA, it is *predictive* of one’s relatives’ DNA, and the closer the relative, the higher the similarity. Thus, by sharing one’s DNA one also shares statistical data about one’s relatives (past, current, and future). Given that one cannot consent to share another individual’s DNA, one ought not to be allowed to freely share a statistical model of another individual’s DNA either, which means that there ought to be limitations on sharing one’s DNA, too. Using the example of DNA and other examples, Carissa Véliz argues that we have a collective interest in privacy and thereby we must also restrict sharing which affects everyone collectively.^33^ My point here is somewhat different; I am arguing that because of the prowess of information aggregation, we cannot avoid infringing upon others’ privacy when we collectively contribute to largescale information collection. Hence, by using a non-MDPP, users violate non-users’ (right to) privacy as well, because the information they share also tells us a lot about other individuals.^34^ While I am not convinced that privacy should be extended to a collective interest, it is clear that the overall informational harms and risks are partly collective. Hence, it is not merely a question of whether we affect others’ privacy when trading our individual information, it is also a question of whether the overarching problems raised in the second section outweigh any *pro tanto* rights or permission to share information.

Again, we have to consider the balancing act between different interests (the right to consent and the consequences for others). Using Hansson’s framework, we can ask whether this type of risk-taking would be equitable. The problem is that if we are allowed to expose non-users—or simply other people—to information-based risks by allowing the usage of information about us, then why should there be any restrictions on information usage *simpliciter*? While the analysis Hansson provides allows for trading different types of risks against other types of risks, there is good reason to think that there should be special restrictions on information-based risks since these risks co-aggregate. That is, the more information that is available, the more information can be predicted, inferred, or de-anonymized.^35^ Informational risks are also special in the sense that they are difficult to mitigate.^36^ There is a form of multidimensional slippery slope argument to worry about here. If risk trading with information and data is allowed in this context, then similar types of information-based risks ought to be allowed in other contexts. Moreover, since information aggregates we have more information jointly than the sum of the information released in each singular case, which means that even if there is a threshold we ought not to pass in a singular context, we would likely pass that threshold because the information made available from all singular contexts would aggregate to surpass that threshold. So, we are *forced* down the slope, even without any norm change, which would be a normal constituent part of a slippery slope argument.

Moreover, we may worry about how information usage in practice may affect and change information distribution norms for the worse. That is, public opinion or individual choices concerning information distribution may change because a more liberal information distribution becomes more common. Hence, this may further exacerbate the problem. There are further problems related to consent, such as whether giving full notice is compatible with giving full consent because of the difficulty of ensuring that individuals are relevantly informed,^37^ which could be further exasperated by information aggregation. 38

All of the above arguments speak in favor of an MDPP and against the right to consent. In summation, we have strong consequence-based arguments in favor of an MDPP. Moreover, the *prima facie* right to consent to information sharing is not satisfied in this case.

## Summation and Concluding Discussion

In this article, I raised some problems with consent agreements for using data and information from non-users of AVs. Given that informed consent practices cannot be applied, I reformulated the problem as a problem of information risk, using Hansson’s conception of an overridable right against risk exposure, arguing that the right against risk exposure was not overridden for the non-users because it was equitable.

Next, I turned to users, arguing that despite their ability to give consent such consent agreements should not be permissible because it is not practically possible to restrict the informational harms to the consenting individuals. Moreover, I pointed out how general problems with informed consent also apply here. These arguments contributed to the conclusion that a maximal data protection policy is the only permissible standard for handling data and information. Hence, it is impermissible to collect or use data and information beyond what is required for safety purposes (or due to other ethical demands).

Finally, I would like to make two forward-looking comments. First, there is certainly a lot to be said about the regulatory implications following from the arguments I have addressed in this paper, and how the arguments cohere with already existing regulations for data protection and AI (such as EU’s GDPR and AI Act). I will not discuss the latter issue, but I will briefly comment on the former. Granted that ethical considerations ought to underpin regulations, we can note that the conclusions from this paper have the benefit of supplying a solution to the contextual limits of ethical guidelines because an MDPP applies the strictest standards in any given context, which also coheres nicely with a call for a global AI ethics.^39^

Second, as I noted in second section, while these arguments are limited to the context of AVs that does not mean that the argumentative model cannot be applied to other cases. Indeed, there is no reason to think that the argument cannot be applied *mutatis mutandis* to other sectors. However, the argument will need to be applied to the given case. There are *prima facie* three considerations that should be considered when shifting the argument to other technologies and sociotechnical contexts. First, the arguments of this paper ultimately depend on consequences that are context-specific (this applies even to the deontological considerations). It is not clear that what is (in)appropriate for transportation is (in)appropriate for all other sectors. Simply put, one needs to consider the contextual variance of the balancing-act between different ethical weights. Second, AVs’ data collection generally includes third-party data, which—even if it occurs in other domains—is simply unavoidable in the cases of AVs. Third, and this applies only to some sectors, there is an important ethical difference in how we shape the *future* of transportation and what changes we should make to, for example, business practices *already in place*.^40^

It is interesting to consider applying the argument to other technologies and sociotechnical contexts. Perhaps the most interesting and fitting case study is not another sector, but, more generally, to look at the Internet-of-things in some given context. For example, it seems *prima facie* clear that there should be strong limits on how data from traditionally private spaces can be used (such as people’s homes); however, those are topics for other papers.

## Data Availability

The manuscript has no associated data.

## Abbreviations

AV: Automated *or* autonomous vehicle
MDPP: Maximal data protection policy
GDPR: The General Data Protection Regulation
LIDAR: Light detection and ranging

## Footnotes

1 In just California, 29 companies with permits reported testing autonomous vehicles on January 1, 2021 (see: https://www.dmv.ca.gov/portal/vehicle-industry-services/autonomous-vehicles/disengagement-reports/). Since then, a lot has happened. For example, Mercedes has achieved SAE level 3 (https://www.motortrend.com/news/2024-mercedes-eq-eqs-us-drive-pilot-quick-drive-review/) and as of June 2024, Waymo’s robottaxi is now fully available to anyone in San Francisco (https://www.theverge.com/2024/6/25/24184814/waymo-waitlist-robotaxi-san-francisco-app-ride).

2 Although there are divergent opinions on when and if AVs will reach full autonomy (level 5), the question I address here will not turn on whether we have vehicles that are driven without human involvement, but whether we have vehicles that have sensorial inputs and access to other data or informational inputs.

3 For an overview, see Hansson et al. (2021).

4 For overviews, see Nyholm (2018a,b).

5 Borenstein et al. (2019); Glancy (2012); Hevelke and Nida-Rümelin (2015); Hansson et al. (2021); Himmelreich (2018); Holstein et al. (2018); Lin (2014); McBride (2016); Mladenovic and McPherson (2016); Nyholm (2018); Ryan (2019); Santoni de Sio (2017); Stone et al. (2020); Wolkenstein (2018).

6 A focused, but still normatively limited discussion is available in Glancy (2012), which from a legal perspective discusses remedies in relation to personal autonomy privacy interests, personal information privacy interests, and surveillance privacy interests. There are also some semi-detailed discussions, for example, in Ryan (2019) and Hansson et al. (2021). Moreover, there are some articles focused in particular on AV-related technologies such as vehicle-to-vehicle communication (see, e.g., Zimmer, 2005). In Vrščaj et al. (2020) there is some empirical data on attitudes to AVs, including privacy issues. Finally, there are several more minor treatments (see fn. 5) as well as technical standards and guidelines (see, e.g., https://www.ieee.org/standards/).

7 Glancy (2012).

8 The focus here is on whether it is permissible to apply the principles of the data economy to the transport economy, but there is no reason why the argument shouldn’t hold for state surveillance as well. Indeed, in repressive regimes, the arguments would be even stronger.

9 It is not unreasonable to think that Alphabet (the parent company of Google) or other similar companies want to expand their business model to the transport sector.

10 See, e.g., Lundgren, 2021b, c; Munch, (2020).

11 Matthews (2010); Lundgren (2020).

12 Lundgren (2020).

13 Lundgren (2020); cf. e.g., Wallace (1999); Ohm (2010); Véliz (2020).

14 van den Hoven (1997); Lundgren (2020); Ohm (2010); Véliz (2020).

15 Deleuze (1992).

16 van den Hoven (1997); Véliz (2020).

17 Lundgren (2020); Véliz (2020).

18 One may think that non-camera-based sensors, such as LIDAR, would resolve some of the problems. While an MDPP certainly must imply certain technical requirements, even LIDAR allows for detailed models of the spatial surroundings (indeed, this is necessary in order for the vehicles to be able to make determinations of whether there are or will be objects in their travel path), so it is not clear whether that would resolve all worries. Nevertheless, a relevant follow-up to this paper would be to discuss precise technical requirements.

19 Hansson et al. (2021, p. 1395).

20 See, e.g., Lundgren (2020), Ohm (2010), and Véliz (2020). See also Zimmer (2005); Glancy (2012); Hansson et al. (2021).

21 See Ohm (2010) and Lundgren (2020). See also Kosinski et al. (2013) for one illustrative model.

22 Lundgren (2020).

23 See, e.g., Lundgren (2020) and Véliz (2020).

24 Hansson et al. (2021) for a brief overview.

25 Stecklow et al. (2023). Relatedly, Selyukh (2013) reported how employees at NSA abused governmental spy tools for private purposes.

26 For my arguments to hold, it is not necessary to settle the precise formulation of such a policy. Indeed, it cannot be fully specified as the formulation of such a policy depends on discussions that I set-aside (e.g., the trade-off between privacy and safety). That is, the MDPP is simply put the maximal data protection policy demands compatible with the ethical balancing considerations I have set aside. If one worries about this, one can read the arguments as simply showing that the type of data use, processing, collection, and creation that is of concern in this paper is impermissible. However, I take it that the arguments in conjunction with the risk of abuse (as discussed in the second section) are sufficiently strong as to also yield a demand for protections against such risks. For a discussion on the contextual nature of information security, see Lundgren and Möller (2019) and Lundgren (2024).

27 Hansson (2003, p. 305).

28 For example, the problem with relying on vague concepts is that they provide imprecise conceptual boundaries. However, my reliance on the underlying normative concepts does not rely on their boundaries. Similarly, we might worry that there is conceptual disagreement regarding how we specify the underlying concepts. However, my arguments do not rely on contested conceptual presumptions. Indeed, the extension of the concepts I use is uncontroversial and motivated through examples building on Hansson (2003).

29 An interesting example is the idea that AV services could partake in the decision about the destination. That is, if a user wants to go shopping for groceries, the AV service could offer a discount if the user travels to a partner store. Hansson et al. (2021, p. 1396) considers such an example.

30 For example, some hold that privacy is important to maintain a social or intimate relationship (e.g., Fried, 1970; Rachels, 1975; Gerstein, 1978), while others think that privacy is about respect for persons or dignity (e.g., Benn, 1984[1971]; Bloustein, 1964).

31 See, e.g., Anderson (2020).

32 Recently, Gustafsson (2021) has argued that classical utilitarianism need not depend on traditional moral aggregation (such as “*The Total Principle*: Outcome X is at least as good as outcome Y if and only if the sum total of well-being is at least as great in X as in Y”, p. 256), but can instead be grounded on principles about best outcomes.

33 Véliz (2020, pp. 75–82).

34 For a recent discussion about the right to privacy and statistical inferences see Munch (2021). See also Lundgren (2021b,c) for a discussion about the right to privacy protecting against substantial risks.

35 Ohm (2010); Lundgren (2020).

36 Although we can use techniques as differential privacy to protect an individual’s anonymity, that does not help to alleviate the overall worry about influencing democratic elections, which in itself is substantial enough to warrant limitations on consequentialist grounds. Differential privacy is a framework to protect user anonymity by introducing statistical noise in a dataset to make it mathematically impossible to re-identify singular individuals in the given dataset based on purely that data (Dwork & Roth, 2014).

37 See, e.g., Nissenbaum (2011) and Solove (2013).

38 Cf. Ohm (2010); Lundgren, 2020).

39 Lundgren (2023a,b); Lundgren et al. (2024).

40 Of course, some think that the whole data economy needs to be radically changed (e.g., Véliz, 2020).
